# Serum bile acids profiles are altered without change of the gut microbiota composition following a seven-day prednisolone therapy in severe alcoholic hepatitis

**DOI:** 10.1080/19490976.2024.2382767

**Published:** 2024-07-30

**Authors:** Damien Esparteiro, Grégory Fouquet, Anoïsia Courtois, Guillaume Jedraszak, Léa Marticho, Mathilde Gourdel, Stéphanie Billon-Crossouard, Mikaël Croyal, Mickaël Naassila, Eric Nguyen-Khac, Ingrid Marcq

**Affiliations:** a GRAP INSERM U1247, Universite de Picardie Jules Verne, Amiens, France; b CHU d’Amiens, Service de Génétique, Amiens, France; c CHU d’Amiens, Service d’Hépato-Gastro-Entérologie, Amiens, France; d CHU Nantes, CNRS, INSERM, BioCore, US16, SFR Bonamy, Nantes Université, Nantes, France; e CRNH-Ouest Mass Spectrometry Core Facility, Nantes, France; f CNRS, INSERM, l’Institut du Thorax, Nantes Université, Nantes, France

**Keywords:** Severe alcoholic hepatitis, prednisolone, corticosteroids, gut microbiota, Lille model, short-chain fatty acids, bile acids

## Abstract

Severe Alcoholic Hepatitis (sAH) is an acute form of liver injury caused by chronic and heavy alcohol drinking. A one-month corticosteroids course is the only sAH reference treatment, and its interactions with the Gut Microbiota (GM), which is a key contributor to liver injury, remain unknown. To evaluate the evolution of the GM in sAH patients, we retrospectively investigated the composition of the GM of 27 sAH patients at the Amiens University Hospital before (D0) and after (D7) a 7-day corticotherapy course using fecal metagenomics sequencing. We also quantified fecal Short-Chain Fatty Acids (SCFA) and fecal and serum Bile Acids (BA), as well as serum Lipopolysaccharide-Binding Protein (LBP). Overall, the community and taxonomical analyses did not reveal any GM evolution between D0 and D7, nor did the SCFA profiles analysis. However, in serum but not fecal samples, the ratio of glyco-conjugated to tauro-conjugated BA was significantly reduced at D7, independently of the response to treatment, while two BA were enriched in non-responder patients. LBP concentration significantly diminished between D0 and D7, which may indicate an improvement of the gut barrier. The stability of the GM of sAH is interesting in the perspective of new treatments based on GM modulation.

## LAY SUMMARY

- There is a gap in the understanding of the effects of corticosteroids on the gut microbiota of severe alcoholic hepatitis patients.
- In this study, the composition of the Gut Microbiota of sAH patients treated with prednisolone remains unchanged after 7 days of prednisolone treatment.
- Short-Chain Fatty Acid profiles are not impacted by the treatment, while Bile Acids profiles change in serum but not in stool samples.
- Responders and non-responders show different lipopolysaccharide-binding protein serum concentration evolution across time, as well as distinct Bile Acid profiles.

## Introduction

Alcohol-related Liver Disease (ALD) refers to a broad spectrum of liver injury resulting from the consumption of alcoholic beverages. Chronic stages of ALD range from steatosis, the reversible accumulation of triglycerides in the liver occurring in 90% of heavy drinkers, to hepatocellular carcinoma.^1^ In addition to the chronic progression of ALD, some individuals may develop severe Alcoholic Hepatitis (sAH), an acute form of liver injury. sAH is a clinical syndrome occurring due to chronic and excessive ethanol consumption. sAH patients usually present with acute-on-chronic liver failure typically with an underlying cirrhotic liver.^2^ As such, the main hallmark of sAH is the sudden onset of jaundice, sometimes alongside extra-hepatic complications such as hepatic encephalopathy or hepatorenal syndrome. Hence, sAH has a poor prognosis, with a mortality rate close to 20% 1 month after presentation.^3^ Combined with alcohol abstinence, one-month corticosteroids (oral prednisolone, 40 mg/day) and five days intravenous N-acetylcysteine courses remain the only reference treatment for sAH.^4,5^ Although prednisolone use is still a matter of debate, in a recent meta-analysis, patients receiving prednisolone had significantly decreased 1-month mortality compared with controls (hazard ratio 0.64, confidence interval 0.48–0.86).^6^ No difference was detected in 6-months mortality, indicating an urgent need for alternative therapies.

On the other hand, human Gut Microbiota (GM) has been extensively studied for the past two decades.^7,8^ GM refers to the rich and diverse community of micro-organisms (including bacteria, archaea, fungi, viruses, and protists) inhabiting the human digestive tract. It is estimated that GM encodes at least 100 times more genes than its human host^9,10^ and plays a role in several metabolic diseases.^11,12^ A significant contribution of the GM to the host homeostasis is achieved through the production and modification of diverse metabolites. Among them, Short-Chain Fatty Acids (SCFA), which are directly produced by the GM, are closely linked to the intestinal epithelium barrier, and can have distal actions on other host organs.^13^ Another family of metabolites closely linked to the GM are Bile Acids (BA). BA are produced and conjugated in the liver, metabolized by the GM, and then reabsorbed in the circulation to be recycled in the liver.^14^ The GM primarily deconjugates BA through bile salt hydrolase production and can also metabolize primary BA into secondary BA.^15^ Among other metabolic pathologies, GM is increasingly recognized as a main actor of ALD pathogenesis.^16^ Indeed, for the same alcohol consumption, GM, alongside other factors, may explain why ALD progresses toward sAH in some individuals while others only present steatosis after years of alcohol drinking.^17,18^

Multiple studies have recently investigated the composition of the sAH GM.^19–22^ These studies report that the GM of sAH patients is dysbiotic, characterized by the loss of microbial diversity, the depletion of commensal and mutualistic microorganisms and the enrichment of pathobionts.^20,22,23^ Preclinical and clinical interventional studies indicate that there is a causative link between GM dysbiosis and liver injury.^17,24^ As intestinal permeability is increased, gut-liver translocation of Microbe-Associated Molecular Patterns (MAMPs) and/or live micro-organisms can occur. This sustained translocation triggers an inflammatory cascade leading to hepatocellular death and liver injury.^25,26^ Preventing this sustained translocation through GM modulation represents a potentially effective way of treating sAH patients, according to recent clinical trials.^27,28^

Treatment of sAH patients with corticosteroids has been extensively documented and debated in the past decades as reviewed elsewhere.^29^ Nonetheless, there is a gap regarding the interactions between prednisolone administration in sAH patients and their GM. We advocate that such interactions cannot be ruled out. While oral prednisolone absorption primarily occurs in the upper gastrointestinal tract, a study of Shaffer et al. conducted on Crohn’s disease patients reports that it may reach the colon and be recovered in feces.^30^ Thus, prednisolone may locally act upon the gut barrier equilibrium between luminal and mucosal MAMPs, and the immune system in the digestive tract and the *lamina propria*. Moreover, the GM is recognized as a key factor in the metabolism of xenobiotics,^31,32^ indicating that direct GM-prednisolone interactions may occur in the gut. In addition, prednisolone may have a distal effect on the gut in spite of a low intestinal bioavailability due to systemic anti-inflammatory effects.

Whether GM can alter prednisolone kinetics, directly and/or indirectly, is unknown. And, conversely, whether prednisolone interacts with the GM can be of clinical interest in the current context of developing sAH treatments based on GM modulation. Interestingly, a recent study did not report any pharmacokinetic differences in prednisolone absorption between prednisolone responders and prednisolone non-responders, which indicates that other factors are involved in the response variation in sAH patients.^33^ Such a factor could be the composition of the GM. Recent research from Shukla et al. on a preclinical model suggests that prednisolone contributes to liver injury through worsening GM dysbiosis and impacting the gut-liver axis.^34^ Another study reported that chronic daily oral administration of prednisone (a prednisolone prodrug) for 6 weeks significantly altered GM composition and metabolite production in rats.^35^

The aim of this pilot study was to investigate the evolution of the GM composition in sAH patients undergoing a prednisolone course. We hypothesized that the GM of sAH patients is impacted by the therapy, and that an improvement in liver injury can be paralleled by GM shifts. We also expected additional factors such as antibiotics exposure, alcohol withdrawal, hospital diet and environment to trigger GM composition shifts. Stool samples from sAH patients were obtained before the initiation of the prednisolone therapy (D0) and after a week of treatment (D7) in order to study the evolution of the GM composition through metagenomics sequencing. In an attempt to gain insight into any functional GM change, we furthermore quantified through mass spectrometry SCFA and BA.

## Material and methods

### Study design

This study has been approved by the national hospital authorities (clinical trial identifier NCT06159244, authorization “Hors loi Jardé” “PI2023_843_0123” granted by “CHU Amiens-Picardie”). This is a comparative, descriptive, retrospective, and monocentric study conducted on samples from patients hospitalized in the Hospital of Amiens, France.

The inclusion criteria were age between 18 and 75, written informed consent, hospital presentation for liver failure due to chronic and excessive consumption of alcohol (≥50 g/d for at least the last 3 months) and a Maddrey Discriminant Function (MDF) ≥32.

Clinical and biological parameters of each sAH patient were recorded, both at D0 and D7. When they were available, stool samples were collected at both timepoints, thoroughly homogenized, aliquoted, and stored at −80°C within 2 hours following defecation in the Biobank of the University Hospital of Amiens. All mandatory laboratory health and safety procedures have been complied when the experiments were carried out.

### Short-chain fatty acids measurements

Quantification of SCFA was performed using Gas Chromatography-Mass Spectrometry as described previously^36^ by processing approximately 50 mg of stool samples.

### Bile acids measurements

Quantification of BA in stool and serum samples was performed using High-Performance Liquid Chromatography – Mass Spectrometry as described previously^37^ by processing either 50 mg of stool samples or 250 µL of serum samples.

### Lipopolysaccharide-binding protein measurement

Lipopolysaccharide-Binding Protein (LBP) was quantified in serum samples using an Enzyme-Linked ImmunoSorbent Assay kit (E-EL-H6108, Elabscience, Houston, USA). Serum samples were diluted 10,000-fold and the quantification was performed according to the manufacturer’s instructions.

### DNA processing from stools

DNA was isolated from stool samples using the QIAamp Fast DNA Stool Mini Kit (Qiagen, Hilden, Germany) following the manufacturer’s instructions with slight modifications: the chemical lysis was reinforced by adding 20 µL of mutanolysin (1 unit/µL, MSD, Kenilworth, USA) and 10 µL of lysozyme (10 mg/mL, Sigma-Aldrich, St-Louis, USA) to 1 mL InhibitEX buffer on a tube containing 220 mg of stool and 300 mg of zirconium beads. A mechanical lysis step was then carried out with three rounds of 30 s of lysis at maximum speed with a Minilys homogenizer (Bertin Technologies, Montigny, France) spaced with rest on ice. Upon protocol completion, DNA extracts were stored at −20°C until further processing.

### Metagenomics sequencing

Metagenomics libraries were prepared and purified according to the Illumina DNA Prep protocol (Illumina, San Diego, USA). DNA concentrations were recorded (Qubit 3 Fluorometer, Thermo Fisher Scientific, Waltham, USA) before library preparation, and once again before library pooling, enabling us to normalize the libraries. After library pooling and final dilutions, DNA sequencing was performed with an Illumina NextSeq 550 device using a paired-end read length of 2 × 150 bases (NextSeq 500/550 High Output Kit v2.5).

### Metagenomics analyses

Demultiplexed fastq-files of forward and reverse reads were obtained from the sequencing device. Reads were first processed using KneadData (version 0.12.0) for cleanup with default parameters. KneadData’s pipeline integrates Trimmomatic (version 0.39), which was used to remove reads with a length <50 bp.^38^ Tandem Repeats Finder (TRF) was employed to remove nucleotide tandems.^39^ Bowtie2 v2.2.5^40^ was used to remove human reads by mapping the sequences against the human genome (GRCh38, Genome Reference Consortium Human Build 38 patch release 7). A total of 808,956,502 ready-to-use reads were obtained, with a mean and a standard deviation of 14,980,676 and 13,555,347 reads per sample respectively. Reads were then processed with MetaPhlAn 4.0.1^41^ in order to obtain microbial absolute abundance tables from gene marker counts. These tables were merged and then imported into the R environment for further processing using the phyloseq package.^42^ The sequencing depth was largely sufficient to detect most of the microbial species in every sample according to the rarefaction procedure (Figure S1).

### Statistical analyses

Statistical analyses were conducted in the R environment. Values are expressed as means and SD. The Shapiro–Wilk test was used to assess the data normal distribution, and the Levene test was employed to verify homoscedasticity. Clinical variables, α-diversity metrics, SCFA, BA and LBP concentrations were compared between D0 and D7 using paired two-sample Student or Wilcoxon tests depending on the data distribution. In a secondary analysis, the distribution of these variables was compared between Prednisolone Responders (PR) and Prednisolone Non-Responders (PNR) at D0 and D7 using ANOVA or Kruskal–Wallis tests, with post-hoc analysis using Tukey’s or Dunn’s tests respectively, depending on the variable distribution. Baseline clinical variables were also compared between PR and PNR using unpaired Student or Wilcoxon tests. The effects of several variables on GM β-diversity were assessed with the permutational multivariate analysis of variance (PERMANOVA) test with 9,999 permutations from the vegan package.^43^ Associations between microbial abundances and individual features were tested using a multivariate linear mixed model in MaAsLin 2. Microbial abundances were normalized using total sum scaling then they were log-transformed^44^ and analyzed using linear models. Only the features with a prevalence of 10% and above and with an absolute read count equal to or higher than 500 were tested, and the obtained *p*-values were corrected for multiple comparisons using the Benjamini–Hochberg procedure for false discovery rate adjustment. Such a correction was also applied after performing post-hoc tests. The correlation between Lille score and LBP concentration or the temporal variation of LBP concentration was assessed by calculating Spearman r. *P*-values of 0.05 and corrected *P*-values of 0.05 were considered to be statistically significant.

## Results

### Patients characteristics

**Figure 1.**
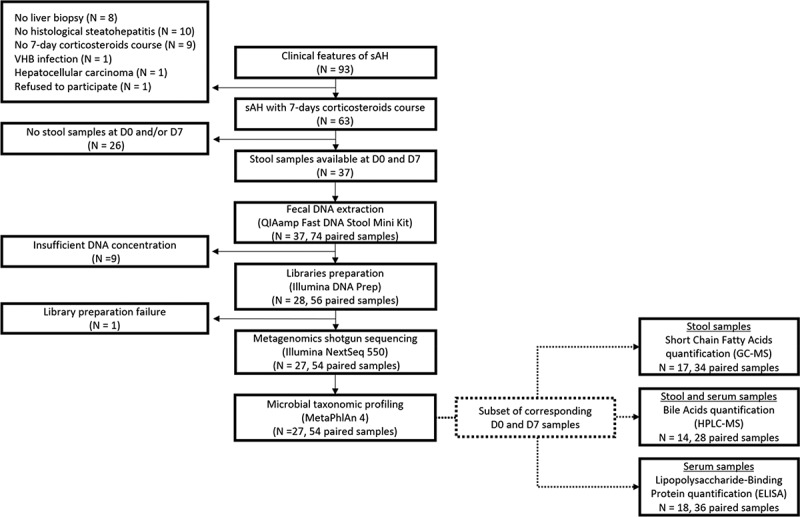
Schematic representation of the study design.

Twenty-seven patients with a diagnosis of sAH confirmed by a liver biopsy, displaying a complete 7-day prednisolone course, and for which stool samples were available at D0 and D7 were included (Figure 1). The clinical characteristics of these patients at D0 are presented in Table 1. The average age of the population was 51.9 years, with 66.7% of the patients being male, and a mean Body Mass Index of 28.3. As expected, every patient displayed jaundice, the main clinical hallmark of sAH, with a liver failure confirmed by an average Model for End-Stage Liver Disease score of 24.4 and an average Maddrey Discriminant function of 60.4. Most (96.3%) of the patients received an additional 5-day course of N-acetylcysteine between D0 and D7. Antibiotics usage was frequent: 29.6% of the population received antibiotics the week before D0, while 33.3% received antibiotics between D0 and D7, highlighting the important infectious vulnerability of sAH patients. Prognosis assessment at D7 using Lille model allowed the classification of 16 patients as Prednisolone Responders (PR, Lille score ≤ 0.45) and 11 patients as Prednisolone Non-Responders (PNR, Lille score > 0.45). PR patients were on average younger than PNR patients (46.9 vs 59.1 years, *p* = .001), as was expected since age is included in Lille score calculation. PR patients’ survival was significantly higher than PNR at 3 months (100.0% vs 54.5%, *p* = .007) and 6 months (100.0% vs 40.0%, *p* = .002), which confirms the ability of Lille model to predict sAH prognosis. No difference was found between PR and PNR regarding other clinical characteristics.

For each step, the N number of patients included is represented, with the associated number of samples when applicable.

**Table 1.** Characteristics of the sAH patients included in the study.

	sAH (*n* = 27)	PR (*n* = 16)	PNR (*n* = 11)	*P*-value
Age (years)	51.9 ± 10.0	46.9 ± 8.4	59.1 ± 7.5	**0.001**
BMI (kg/m^2^	28.3 ± 4.5	27.7 ± 5.3	29.4 ± 2.0	0.520
Male gender (%)	18 (66.7)	11 (68.8)	7 (63.6)	1.000
Estimated ethanol intake (g/day)	109.9 ± 102.0	123.5 ± 110.3	91.8 ± 92.8	0.545
Antibiotics usage the week before D0, *n* (%)	8 (29.6)	4 (25.0)	4 (36.4)	0.675
Antibiotics usage the week before D7, *n* (%)	9 (33.3)	5 (31.3)	4 (36.4)	1.000
N-Acetylcystein usage, n (%)	26 (96.3)	15 (93.8)	11 (100.0)	1.000
**Clinical symptoms**				
Jaundice, *n* (%)	27 (100.0)	16 (100.0)	11 (100.0)	1.000
Ascites, *n* (%)	16 (59.3)	8 (50.0)	8 (72.7)	0.427
Hepatic encephalopathy, *n* (%)	5 (18.5)	4 (25.0)	1 (9.1)	0.619
Gastrointestinal hemorrhage, *n* (%)	2 (7.4)	1 (6.2)	1 (9.1)	1.000
**Clinical scores**				
MDF	60.4 ± 17.4	56.1 ± 15.5	66.5 ± 18.8	0.132
MELD	24.4 ± 5.0	24.1 ± 5.4	24.8 ± 4.4	0.765
Lille	0.4 ± 0.3	0.2 ± 0.1	0.8 ± 0.2	**<0.001**
**Liver histology**				
Mallory-Denk bodies, *n* (%)	21 (77.8)	13 (81.2)	8 (72.7)	0.662
Hepatocyte ballooning, *n* (%)	23 (85.2)	13 (81.2)	10 (90.9)	0.624
Steatosis, *n* (%)	22 (81.5)	15 (93.8)	7 (63.6)	0.125
Cirrhosis, *n* (%)	24 (88.9)	13 (81.2)	11 (100.0)	0.248
**Survival**				
at 1 month, *n* (%)	25 (92.6)	16 (100.0)	9 (81.8)	0.157
at 3 months, *n* (%)	21 (80.8)	15 (100.0)	6 (54.5)	**0.007**
at 6 months, *n* (%)	17 (73.9)	13 (100.0)	4 (40.0)	**0.002**
at 1 year, *n* (%)	11 (55.0)	8 (72.7)	3 (33.3)	0.175

sAH diagnosis was confirmed with a trans-jugular liver biopsy. Values are expressed in number (percentage) or mean ± standard-deviation. Fisher tests were used for categorical variables; two-sample t-tests were used for normally distributed continuous variables and Wilcoxon rank tests were used for non-normally distributed continuous variables. *p*-values <.05 are bolded and statistically significant. Abbreviations: BMI: Body Mass Index; D0: Day 0 of the treatment; D7: Day 7 of the treatment; MDF, Maddrey Discriminant Function; MELD, Model for End-Stage Liver Disease; PNR, Prednisolone Non-Responder; PR, Prednisolone Responder.

### Prednisolone administration is associated with changes in circulating biochemical markers

Circulating biochemical markers evolution between D0 and D7 is represented in Table 2. After 7 days of treatment, a significant diminution of C-reactive protein was observed (36.4 µmol/L vs 24.8 µmol/L, *p* < .001, paired Wilcoxon), in parallel with improvements of alanine aminotransferase (50.8 IU/L vs 67.8 IU/L, *p* < .001, paired Wilcoxon) and prothrombin time (39.3% vs 41.7%, *p* = .016, paired Student). This phenomenon is likely explained by the reduction in inflammation due to corticosteroids that translated for most but not all patients with an improvement in liver function. Interestingly, shifts were observed in leukocytes counts. White cell counts significantly increased over time (10.1 ×10^9^ cells per mL vs 12.3 × 10^9^ cells per mL, *p* < .001, paired Wilcoxon) with an elevation of the relative abundance of neutrophils (73.8% vs 76.3%, *p* = .028, paired Student) over that of monocytes (9.6% vs 7.9%, *p* < 0.001, paired Wilcoxon) and basophils (0.6% vs 0.3%, *p* = .021, paired Wilcoxon). We further compared the evolution of these variables between PR and PNR (Table S1). Unsurprisingly, the increase of prothrombin time and the decrease of total bilirubin concentration were characteristics of PR but not PNR patients. Interestingly, a diminution in C-reactive protein levels was numerically observed for PR patients, but not for PNR.

**Table 2.** Evolution of blood biochemical markers between D0 and D7 of treatment.

	D0 (*n* = 27)	D7 (*n* = 27)	*P*-value
Albumin (g/L)	25.5 ± 5.5	27.2 ± 7.2	0.893
Alkaline phosphatase (IU/L)	159.1 ± 35.6	178.7 ± 83.8	0.746
Alanine aminotransferase (IU/L)	50.8 ± 25.6	67.8 ± 33.0	**<0.001**
Aspartate aminotransferase (IU/L)	122.9 ± 73.8	129.6 ± 64.3	0.684
Creatinin (µmol/L)	78.8 ± 76.5	68.4 ± 37.4	0.867
C-Reactive protein (mg/L)	36.4 ± 26.1	24.8 ± 15.5	**<0.001**
γ-glutamyltransferase (IU/L)	234.1 ± 177.3	215.0 ± 138.5	0.296
Prothrombin time (%)	39.3 ± 9.9	41.7 ± 11.3	**0.016**
Total bilirubin (µmol/L)	234.1 ± 135.4	214.0 ± 132.7	0.186
White cell count (x10^9^/L)	10.1 ± 8.9	12.3 ± 9.2	**<0.001**
Lymphocytes (% of white cell count)	14.0 ± 6.5	13.7 ± 7.9	0.396
Monocytes (% of white cell count)	9.6 ± 2.7	7.9 ± 2.5	**<0.001**
Polymorphonuclear basophils (% of white cell count)	0.6 ± 0.8	0.3 ± 0.6	**0.021**
Polymorphonuclear eosinophils (% of white cell count)	1.8 ± 1.2	1.6 ± 1.2	0.348
Polymorphonuclear neutrophils (% of white cell count)	73.8 ± 8.5	76.3 ± 10.2	**0.028**

Values are expressed in mean ± standard deviation. Paired t-tests were used for comparing normally distributed continuous variables and paired Wilcoxon tests were used for comparing non-normally distributed continuous variables. *P*-values <.05 are bolded and statistically significant. Abbreviations: D0: Day 0 of the treatment; D7: Day 7 of the treatment.

### Fecal microbiota diversity and taxonomy are independent to time to sampling

**Figure 2.**
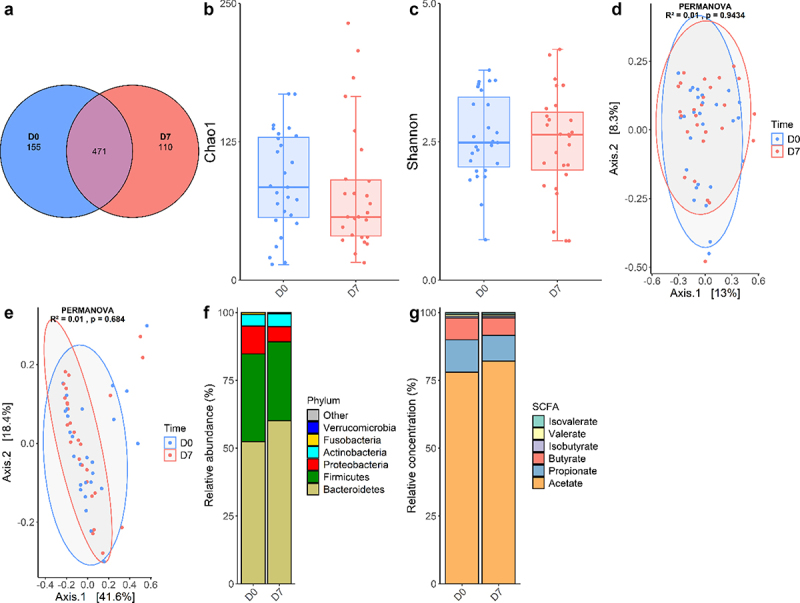
Analysis of the GM of sAH patients at D0 and D7.

After the shotgun metagenomics sequencing of 54 paired stool samples DNA extracts (27 samples at D0 and 27 samples at D7), clean reads were taxonomically assigned using MetaPhlAn 4, an algorithm that detects microbial marker genes in sequencing reads. A total of 736 species were detected, with a mean of 84.5 species detected per sample. While 471 species were detected at both timepoints, 155 species were only detected at D0, and 110 only at D7 (Figure 2(a)). This slightly decreased value at D7 could be explained by a reduced exposure to transitory micro-organisms in the hospital environment between D0 and D7. Indeed, most of the species specifically detected at only one timepoint belonged to the rare biosphere and were only detected in a small proportion of individuals (Tables S2 and S3). The species richness was evaluated using the Chao1 index (Figure 2(b)), which remained unchanged between D0 and D7 (88.5 vs 80.5, *p* = .254, paired Wilcoxon test). Similarly, Shannon diversity and evenness index (Figure 2(c)) remained stable over time (2.6 vs 2.5, *p* = .574, paired t-test). β-diversity was then evaluated with both Bray-Curtis and Weighted UniFrac metrics. First, the distribution of those metrics was analyzed using a permutational analysis of variance (PERMANOVA) that accounts for the effects of “Time” alone. In this analysis, Time variable did not significantly explain the distribution of the Bray-Curtis (*p* = .943, Figure 2(d)) or Weighted UniFrac metrics (*p* = .684, Figure 2(e)). To determine the variables explaining the composition of the GM, the effects of Time, Response, Sex, Age, Antibiotics, and Individual variables on Bray-Curtis index were simultaneously evaluated. 1.3% (*p* = .306) of the distribution was explained by the time variable, 2.0% (*p* = .014) was explained by the Response variable, 2.9% (*p* < .001) by the Sex variable, 2.0% (*p* = .015) by the Age variable, 3.7% (*p* < .001) by the Antibiotics variable, and finally 59.1% (*p* < .001) by the Individual variable. Interestingly, when analyzing the weighted UniFrac metric accounting for the same variables, only the Antibiotics (7.8%, *p* < .001) and Individual (53.3%, *p* = .003) variables significantly explained the distribution of the beta-diversity metric, while no association was found for Response (1.2%, *p* = .120). The discrepancy between the results for those two metrics can be attributed to Weighted UniFrac accounting for phylogenetic proximity. In other words, the GM composition is partially explained by the responder status only when not accounting for phylogenetic proximity of the micro-organisms, which suggests that the micro-organisms differentially present between PR and PNR are phylogenetically close. Thus, at the level of the community, GM composition is indistinguishable at D0 and D7, and is mostly explained by individual characteristics.

Shared and unique species are displayed with a Venn diagram (a). Species richness Chao1 index was compared using paired Wilcoxon test (*p* = .254) (b) and diversity Shannon index was compared using paired t-test (*p* = .574) (c). GM composition is represented using Principal Coordinate Analyses based on the β-diversity indexes of Bray-Curtis (d) and weighted UniFrac (e), and compared between D0 and D7 using Permutational Multivariate Analysis of Variance (PERMANOVA). Average relative abundances of bacterial phyla are represented in (f). Relative Short-Chains Fatty Acids concentrations in stool samples were calculated (g). In figures (b) and (c), horizontal lines represent median values, boxes represent the interquartile range, whiskers represent 1.5 times the interquartile range.

Then, the analyses of microbial communities were completed with taxonomic analyses. Average relative abundances of the main bacterial phyla were calculated (Figure 2(f)). As expected, Bacteroidetes and Firmicutes represented more than 80% of the GM. Proteobacteria and Actinobacteria encompassed the remaining fraction of the diversity, while Verrucomicrobia and Fusobacteria abundances were negligible. Interestingly, GM composition shows an important inter-individual variability that does not seem related to the time of sampling (Figure 2). For instance, although Proteobacteria and Actinobacteria were overall minor phyla, for some individuals they accounted for most of the GM composition. In an attempt to identify taxa with significant abundance variation between D0 and D7, we conducted a multivariate mixed linear model analysis with MaAsLin 2 at the species level (Table S4). Adjusting for Sex, Age, Antibiotics, Response as fixed effects and Individual as random effect, there was no significant difference between D0 and D7 or between PR and PNR for any species. One species, *Senegalimassilia anaerobia*, was significantly depleted in samples obtained after a recent antibiotics course (q < 0.001), although this species was only detected in seven samples. Similar analyses did not reveal any significant change between D0 and D7 at the phylum, family and genus levels (data not shown). Taken together, these results indicate that GM composition is minimally, if at all, influenced by the sampling timepoint and is primarily dependent on individual factors.

### Serum but not fecal metabolites are linked to time to treatment and prognosis

SCFA were quantified in 34 paired stool samples from 17 sAH patients using GC-MS (Figure 2(g)). The total SCFA concentration did not change between D0 and D7 (105.8 µmol/g vs 129.9 µmol/g, *p* = .119, paired t-test). Relative concentrations of acetate, butyrate, propionate, isobutyrate, valerate and isovalerate remained unchanged between D0 and D7, even when separating PR and PNR patients (Table S5).

BA were then quantified in a subset of 28 paired stool samples (Table 3) and 28 paired serum samples (Table 4) using HPLC-MS. No difference was observed between D0 and D7 in the stool samples for any BA. In serum samples, the total BA concentration did not vary by time (47.9 µg/mL at D0 vs 52.9 µg/mL at D7, *p* = .626). However, the glyco-conjugated to tauro-conjugated BA (G/T) ratio significantly decreased between D0 and D7 (1.4 to 1.1, *p* = .030, paired Wilcoxon). This evolution was explained both by a decrease in the proportion of glyco-conjugated BA (from 53.6% to 45.4%, *p* < .001, paired t-test), and by an increase in the proportion of tauro-conjugated BA (from 44.2% to 49.9%, *p* = .087, paired t-test. We then compared these shifts between PR and PNR patients (Table 5). While the evolution of the G/T ratio described previously was similar for both groups, a trend to an overall difference was observed for the relative concentration of glyco-conjugated deoxycholic acid (Kruskal–Wallis H = 6.673, *p* = 0.083) with this BA being detected only in serum samples of PR, although in a minor proportion of samples and with a low abundance. Variance analysis indicates a significant variation of the tauro-conjugated chenodeoxycholic acid (ANOVA F = 5.650, *p* = 0.004), with the post-hoc analysis revealing a trend to a lower concentration of this BA in PR patients at D0 (22.2% vs 33.1%, Dunn’s test, *p* = .073). This observation was significant at D7 (25.5% vs 37.9%, Dunn’s test, *p* = .035). Likewise, tauro-conjugated ursodeoxycholic acid relative abundance was overall significantly different between groups (Kruskal–Wallis H = 9.99, *p* = .019), with D0 relative concentration being significantly more reduced in PR (0.2% vs 0.6%, Dunn’s test, *p* = .023). No between-group difference was observed for any stool BA in this secondary analysis (Table S6).

**Table 3.** Evolution of stool samples bile acids concentrations between D0 and D7 of treatment in sAH patients.

	D0 (*n* = 14)	D7 (*n* = 14)	*P*-value
**Total bile acids (µg/g)**	113.7 ± 155.2	94.5 ± 128.4	0.808
**Primary bile acids (% of total)**	59.8 ± 37.0	58.4 ± 38.9	0.358
**Free bile acids (% of total)**	90.1 ± 12.5	95.1 ± 5.6	0.296
CA (% of total)	30.9 ± 27.5	35.9 ± 32.2	0.715
CDCA (% of total)	19.7 ± 11.4	17.9 ± 13.5	0.621
DCA (% of total)	14.2 ± 15.1	15.4 ± 18.1	0.969
HDCA (% of total)	0.8 ± 1.6	0.5 ± 1.2	0.402
LCA (% of total)	19.5 ± 24.2	18.7 ± 23.7	0.626
UDCA (% of total)	5.1 ± 4.8	6.8 ± 10.8	0.889
**Glyco-conjugated bile acids (% of total)**	9.9 12.5	4.9 ± 5.6	0.296
CA (% of total)	2.3 ± 4.3	0.8 ± 2.3	0.353
CDCA (% of total)	6.9 ± 8.7	3.9 ± 3.8	0.241
DCA (% of total)	0.2 ± 0.5	0.2 ± 0.5	0.933
HDCA (% of total)	0.0 ± 0.0	0.0 ± 0.0	–
LCA (% of total)	0.3 ± 0.8	0.0 ± 0.2	0.181
UDCA (% of total)	0.1 ± 0.4	0.0 ± 0.0	1.000

Values are expressed in mean ± standard deviation. Paired two-sample t-tests were used for comparing normally distributed continuous variables and paired Wilcoxon rank tests were used for comparing non-normally distributed continuous variables. Abbreviations: CA: Cholic Acid; CDCA: Chenodeoxycholic Acid; DCA: Deoxycholic Acid; D0: Day 0 of the treatment; D7: Day 7 of the treatment; HDCA: Hyodeoxycholic Acid; LCA: Lithocholic Acid; UDCA: Ursodeoxycholic Acid.

**Table 4.** Evolution of serum bile acids concentrations between D0 and D7 of treatment for sAH patients.

	D0 (*n* = 14)	D7 (*n* = 14)	*P*-value
**Total bile acids (µg/mL)**	47.9 ± 28.2	52.9 ± 23.1	0.626
**Primary bile acids (% of total)**	96.0 ± 4.7	95.9 ± 4.8	0.683
**Free bile acids (% of total)**	2.3 ± 3.4	4.7 ± 9.5	0.808
CA (% of total)	0.4 ± 0.4	1.4 ± 3.0	0.761
CDCA (% of total)	1.3 ± 2.0	2.5 ± 4.9	0.808
DCA (% of total)	0.3 ± 1.0	0.4 ± 0.9	1.000
HDCA (% of total)	0.0 ± 0.0	0.1 ± 0.1	0.328
LCA (% of total)	0.1 ± 0.1	0.1 ± 0.2	0.529
UDCA (% of total)	0.2 ± 0.2	0.3 ± 0.5	0.965
**Glyco-conjugated bile acids (% of total)**	53.6 ± 10.2	45.4 ± 9.5	**0.001**
CA (% of total)	16.6 ± 8.5	14.2 ± 7.5	0.186
CDCA (% of total)	35.1 ± 12.3	29.6 ± 10.7	**0.032**
DCA (% of total)	0.7 ± 1.6	0.5 ± 1.0	1.000
HDCA (% of total)	0.0 ± 0.0	0.0 ± 0.0	–
LCA (% of total)	0.2 ± 0.2	0.2 ± 0.3	0.359
UDCA (% of total)	1.0 ± 1.0	0.8 ± 0.9	0.845
**Tauro-conjugated bile acids (% of total)**	44.2 ± 11.9	49.9 ± 15.0	0.087
CA (% of total)	15.7 ± 9.9	17.4 ± 12.0	0.194
CDCA (% of total)	26.8 ± 8.8	30.8 ± 10.4	0.126
DCA (% of total)	0.7 ± 1.6	0.7 ± 1.2	1.000
HDCA (% of total)	0.3 ± 0.4	0.4 ± 0.4	0.442
LCA (% of total)	0.1 ± 0.1	0.1 ± 0.2	0.965
UDCA (% of total)	0.4 ± 0.3	0.4 ± 0.3	0.410

Values are expressed in mean ± standard deviation. Paired two-sample t-tests were used for comparing normally distributed continuous variables and paired Wilcoxon rank tests were used for comparing non-normally distributed continuous variables. Abbreviations: CA: Cholic Acid; CDCA: Chenodeoxycholic Acid; DCA: Deoxycholic Acid; D0: Day 0 of the treatment; D7: Day 7 of the treatment; HDCA: Hyodeoxycholic Acid; LCA: Lithocholic Acid; UDCA: Ursodeoxycholic Acid.

**Table 5.** Evolution of serum bile acids concentrations for PR and PNR patients between D0 and D7 of treatment.

	PR (*n* = 8)		PNR (*n* = 6)		Test	
	D0	D7	D0	D7	Statistic	*P*-value
**Total bile acids (µg/mL)**	53.0 ± 23.2	43.9 ± 17.4	41.2 ± 34.9	65.0 ± 25.6	F = 1.143	0.352
**Primary bile acids (% of total)**	94.6 ± 5.8	94.6 ± 6.1	97.8 ± 1.5	97.7 ± 1.4	H = 1.113	0.774
**Free bile acids (% of total)**	3.1 ± 4.4	7.7 ± 11.9	1.2 ± 0.7	0.8 ± 0.3	H = 1.866	0.601
CA (% of total)	0.4 ± 0.5	2.3 ± 3.9	0.3 ± 0.3	0.1 ± 0.1	H = 3.694	0.296
CDCA (% of total)	1.7 ± 2.5	4.0 ± 6.2	0.7 ± 0.6	0.4 ± 0.2	H = 0.679	0.878
DCA (% of total)	0.5 ± 1.3	0.7 ± 1.2	0.0 ± 0.0	0.0 ± 0.0	H = 5.422	0.143
HDCA (% of total)	0.0 ± 0.1	0.1 ± 0.1	0.1 ± 0.0	0.1 ± 0.1	H = 3.698	0.296
LCA (% of total)	0.1 ± 0.1	0.2 ± 0.3	0.0 ± 0.1	0.0 ± 0.1	H = 2.832	0.418
UDCA (% of total)	0.2 ± 0.2	0.4 ± 0.6	0.1 ± 0.1	0.1 ± 0.1	H = 2.433	0.487
**Glyco-conjugated bile acids (% of total)**	55.6 ± 11.0	46.7 ± 10.3	50.9 ± 9.2	43.7 ± 8.8	F = 1.920	0.153
CA (% of total)	19.3 ± 9.0	14.9 ± 8.8	13.1 ± 6.8	13.3 ± 6.2	F = 0.941	0.436
CDCA (% of total)	33.7 ± 13.8	29.7 ± 13.8	37.1 ± 11.1	29.5 ± 5.6	F = 0.595	0.624
DCA (% of total)	1.2 ± 1.9	0.9 ± 1.2	0.0 ± 0.0	0.0 ± 0.0	H = 6.673	0.083
HDCA (% of total)	0.0 ± 0.0	0.0 ± 0.0	0.0 ± 0.0	0.0 ± 0.0	–	–
LCA (% of total)	0.3 ± 0.2	0.3 ± 0.4	0.1 ± 0.1	0.1 ± 0.1	H = 4.078	0.253
UDCA (% of total)	0.0 ± 0.0	0.0 ± 0.0	0.3 ± 0.6	0.0 ± 0.0	H = 3.667	0.300
**Tauro-conjugated bile acids (% of total)**	41.3 ± 13.5	45.6 ± 17.7	48.0 ± 9.0	55.5 ± 8.7	F = 1.346	0.283
CA (% of total)	17.4 ± 12.4	18.2 ± 15.5	13.5 ± 5.3	16.3 ± 5.8	F = 0.214	0.886
CDCA (% of total)	22.2 ± 7.9^a^	25.5 ± 9.0^a^	33.1 ± 5.6^ab^	37.9 ± 8.0^b^	F = 5.651	**0.004**
DCA (% of total)	0.9 ± 2.0	1.3 ± 1.4	0.5 ± 1.1	0.0 ± 0.0	H = 4.358	0.225
HDCA (% of total)	0.4 ± 0.4	0.3 ± 0.2	0.3 ± 0.3	0.6 ± 0.5	H = 2.291	0.514
LCA (% of total)	0.1 ± 0.1	0.2 ± 0.2	0.1 ± 0.1	0.1 ± 0.1	H = 2.053	0.562
UDCA (% of total)	0.3 ± 0.2^ab^	0.2 ± 0.1^a^	0.5 ± 0.3^ab^	0.6 ± 0.3^b^	H = 9.986	**0.019**

Values are presented as mean ± standard-deviation. *p*-values <0.05 are bolded and statistically significant. Depending on the variable distribution, ANOVA F or Kruskal-Wallis H statistics were calculated. Exponential notations represent significant differences as determined by post-hoc analyses: values sharing the same exponential notation are not statistically different (corrected *p*-value >0.05). Abbreviations: CA: Cholic Acid; CDCA: Chenodeoxycholic Acid; DCA: Deoxycholic Acid; D0: Day 0 of the treatment; D7: Day 7 of the treatment; HDCA: Hyodeoxycholic Acid; LCA: Lithocholic Acid; PNR, prednisolone non-responders; PR, prednisolone responders; UDCA: Ursodeoxycholic Acid.

**Figure 3.**
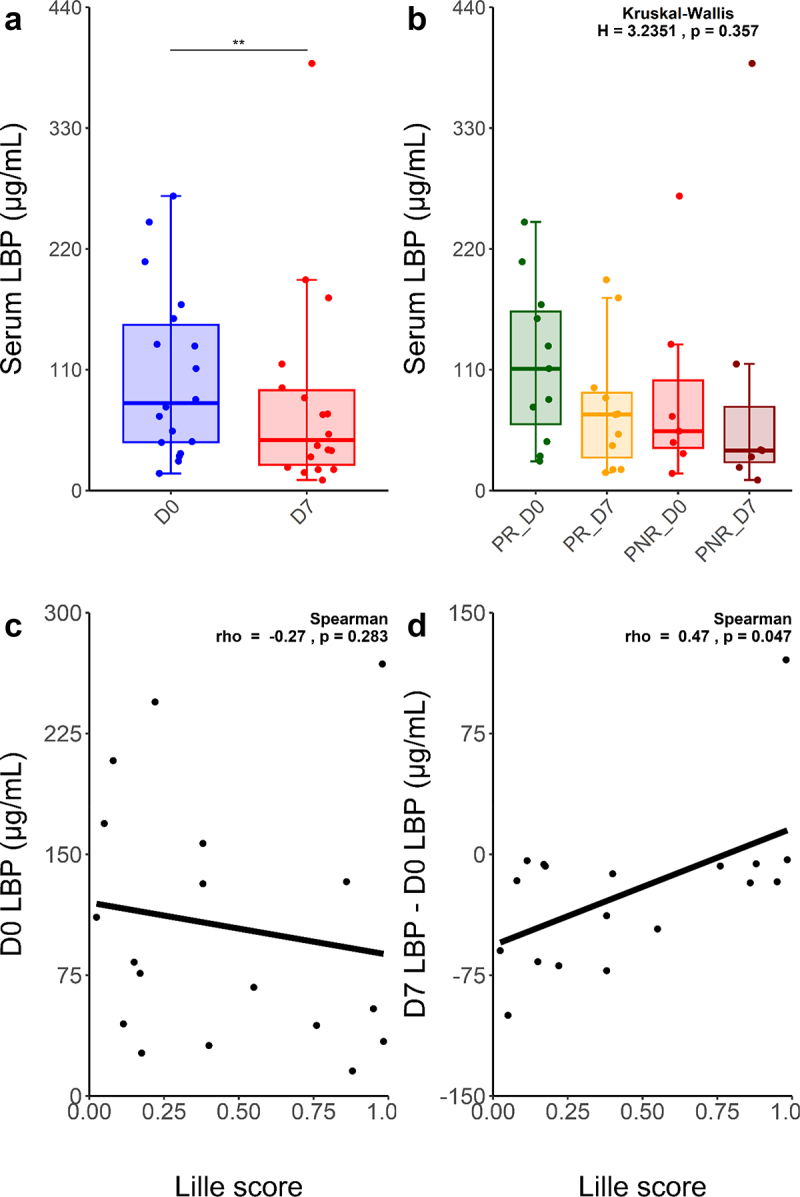
Distinct circulating LBP profiles evolution between PR and PNR sAH patients.

To evaluate the impact of prednisolone on the gut barrier, we quantified serum LBP in 36 samples from 18 sAH patients, both at D0 and D7 (Figure 3(a)). LBP serum concentration significantly decreased between D0 and D7 (105.5 µg/mL vs 81.7 µg/mL, *p* = 0.002, paired Wilcoxon), with PR and PNR profiles being similar (Figure 3(b)). Although Lille score was not correlated with baseline LBP concentration (Figure 3(c)), it was significantly and negatively correlated with the LBP absolute concentration difference between D7 and D0 (Spearman *r* = 0.47, *p* = 0.047) (Figure 3(d)).

Serum LBP concentration is significantly decreased after 7 days of prednisolone treatment (paired Wilcoxon, *p* = 0.002). (a), while both PR and PNR show similar profiles (b). Lille score was not correlated with baseline circulating LBP concentration (Spearman *r* = –0.27, *p* = 0.283) (c) but was significantly and negatively correlated to a decrease of LBP concentration between D0 and D7 (Spearman *r* = 0.47, *p* = 0.047) (d). In (a) and (b), horizontal lines represent median values, boxes represent the interquartile range, whiskers represent 1.5 times the interquartile range. **: *p* < 0.01

## Discussion

Prednisolone therapy, which is the standard of care for sAH management, reduces mortality and improves prognosis in most, but not all, sAH patients. However, its impact on the GM or conversely, the role of the GM in treatment response, remains unknown. To the best of our knowledge, the longitudinal evolution of the GM of sAH patients under prednisolone has not yet been studied. This study aimed to address this gap and to characterize the evolution of the GM composition over a week of prednisolone therapy in sAH patients. The first seven days are significant in the management of sAH because it is only after this duration that the Lille score can be calculated in order to assess the response of sAH patients to treatment and guide further care decisions.

Our hypothesis was that GM composition would shift between D0 and D7 due to prednisolone and additional factors, such as antibiotics exposure. We observed that some species were specifically detected at D0 and other species only at D7, but this difference can be attributed to random chance since these species belonged to the rare biosphere and were detected in only a few samples. In addition, α-diversity, β-diversity and microbial differential abundance analyses did not reveal any significant change in the GM composition after 7 days of prednisolone treatment. As was expected, we observed significant effects of antibiotics exposure, age and sex on the overall distribution of the GM composition.

Since metagenomics sequencing can only shed light on microbial abundances, metabolite analyses were required to assess GM evolution at the functional level. We quantified SCFA in stool samples and BA in stool and serum samples at both timepoints. The comparison of the BA profiles in the stool and serum samples could provide valuable insight into difference in the entero-hepatic BA circulation. While stool SCFA and BA profiles did not change between D0 and D7, we observed in serum samples a significant decrease of the G/T ratio for both PR and PNR, which suggests that it does not necessarily occur due to an improvement of liver function. Primary BA are produced in the liver and may be conjugated with glycine or taurine before being secreted into the gallbladder and excreted in the duodenum to facilitate lipid absorption.^15^ GM micro-organisms can metabolize primary BA in multiple ways, including converting them into secondary BA or deconjugating them, which is in line with the low concentration (<10%) of fecal conjugated BA in our cohort. Thus, the G/T ratio evolution can be explained by at least three factors. First, between D0 and D7, hepatocytes may become more likely to conjugate taurine than glycine, for example due to an increased availability of the former or a decreased availability of the latter.^45^ Second, it is possible that GM interacts differently with BA in the intestines, with an improved ability to deconjugate glyco-conjugated BA over tauro-conjugated BA.^15^ Nevertheless, if GM was causing this shift, it was not due to a change in its composition, since it remained stable between D0 and D7, but rather due to a change in its metabolic activity, which could not be evaluated in this study using metagenomics. Third, this evolution could be explained by a difference in the reabsorption of glyco-conjugated and tauro-conjugated BA by hepatocytes in the entero-hepatic circulation. The implications of the G/T ratio decrease between D0 and D7 are intriguing in light of several literature reports. Harnisch et al.^45^ reported that this ratio was diminished, in comparison to healthy control, in patients with acute respiratory distress syndrome, a pathology in which liver and biliary functions may be impaired, and that survival of these patients was significantly associated with this ratio. This ratio decrease was also observed in other conditions, including viral hepatitis.^46^ This would translate in the present study by a worsening of liver function over the course of treatment, including in PR patients who in contrast demonstrated a better prognosis. Put together, the BA profiles shifts in the serum of sAH patients, in the context of prednisolone use, remain poorly understood and deserve further studies, especially to find out the contribution of the GM.

In order to gain insights into the gut-liver microbial translocation occurring in sAH, we measured LBP in patients’ serum samples obtained at both timepoints. Circulating LBP concentration is highly correlated, under normal circumstances, with circulating lipopolysaccharide concentration, which in turn is closely linked to gut-circulation microbial translocation.^47^ We measured a high baseline mean serum LBP concentration of 105.5 µg/mL, although this measure greatly differed between individuals. In perspective, Gonzalez-Quintela et al. measured a mean serum LBP concentration of 7.5 µg/mL in a healthy Spanish population of 420 individuals,^48^ while Asada et al. reported a close mean of 11.0 µg/mL in a cohort of 2,568 healthy Japanese.^49^ This elevated value in our study probably indicates a baseline impairment of gut barrier in most patients, which was expected since gut-liver translocation is an important source of the liver injury observed in sAH. Furthermore, we observed a significant diminution of this variable between D0 and D7 for most but not all patients. Thus, we investigated a potential link between the Lille score and the LBP concentration decrease between D0 and D7. We found a negative correlation (Spearman *r*= −0.47) between the two variables, indicating that a poor prognosis was associated with the persistence of high levels of circulating LBP. Curiously, this association was not significant when considering the D0 to D7 LBP concentration ratio, which implies that prognosis was improved especially for patients with baseline elevated LBP that importantly diminished between D0 and D7. Two not mutually exclusive causes could explain a decrease of circulating LBP in this context. A first explanation is that this diminution is caused by an improvement of gut barrier function, due to prednisolone treatment and/or alcohol withdrawal. In this case, patients having particularly important diminution of LBP concentration would be the ones having considerable reduction in gut-liver MAMPs translocation. However, since the GM remained stable between D0 and D7, we cannot rule out that the gut barrier remained impaired in the meantime. A second explanation is that prednisolone exerts effects on *LBP* expression directly or indirectly, a notion only supported by two old studies. Schumann et al.^50^ characterized the promoter region of *LBP* and reported that both glucocorticoids and pro-inflammatory cytokines may regulate *LBP* transcription. Another study revealed that the administration of dexamethasone, another glucocorticoid, prevented *LBP* expression induction by LPS or IL-6 in rat hepatocytes.^51^ Hence, a diminution of circulating LBP could be due to prednisolone therapy inhibiting the production of LBP in spite of an unchanged LPS translocation, rather than a diminution of LPS translocation itself. Under both hypotheses, the different LBP concentration evolution between PR and PNR is intriguing. The poorer prognosis of PNR patients could be partially caused by the LBP concentration remaining high over time due to a continuation of gut-liver translocation, but it is also plausible that both phenomena are merely consequences of the lack of prednisolone efficacy. Assessment of the gut barrier evolution across time using other biomarkers is warranted.

While this study primarily aimed at characterizing GM evolution between D0 and D7 of prednisolone therapy using complementary methods, we also had the opportunity to compare, in a small cohort of sAH patients, the composition of the GM between PR and PNR. We found that a small fraction of the GM composition was explained by the response to treatment, although the subsequent differential abundance analysis did not identify species, genus, family or phylum discriminating PNR from PR. This discrepancy might be explained by a lack of statistical power combined with low effect sizes, or by the difference detected analyzing beta-diversity being spread across multiple taxa. The comparison of the baseline GM of PR and PNR patients is currently being carried out in a more substantial cohort and should allow to address this point. Nevertheless, in this study, we also primed the comparison of the baseline SCFA and BA profiles between PR and PNR. While some studies report a significant correlation between severity of sAH and some serum BA,^52,53^ to the best of our knowledge, such correlation with Lille score has not been investigated in past literature. While SCFA profiles remained highly similar in PR and PNR, we observed significant variations in the relative abundance of two tauro-conjugated BA, chenodeoxycholic and ursodeoxycholic acids, which were more important in the serum, but not in the stool samples, of PNR patients. The quantification of these two BA in serum samples of newly hospitalized sAH patients could represent a new biomarker improving the accuracy to predict sAH patient’s prognosis using Lille model.

The stability of the GM over 7 days of treatment is of particular interest in light of the gut-liver axis. Since gut dysbiosis is a main contributor to liver injury, a lack of effects of the reference treatment on GM could explain why prednisolone has not demonstrated long-term benefits on the sAH patient survival. sAH treatment based on GM modulation through Fecal Microbiota Transplantation (FMT) is promising. FMT effects on sAH patients have been increasingly evaluated since the pilot study of Philips et al.^27^ in 2017. Recently, Pande et al.^28^ conducted an open-label randomized clinical trial comparing a daily 7-day FMT course versus a 28-day prednisolone course, and reported a significant improvement of 90-days survival in the FMT arm (75% vs 56.6%) accompanied with a reduced infection rate. Since FMT aims at replacing the dysbiotic sAH GM with a GM from a healthy individual and supposedly eubiotic, the great success rate of this procedure on sAH survival could be explained by its diminishing liver inflammation by acting at the intestinal level, which is not achieved with prednisolone. Recent research suggests that the combination of N-acetylcysteine, prednisolone and FMT may represent a new standard of care to treat sAH and as such deserves further clinical trials.

The main limitation of this study is the presence of potential confounding factors. First, the effects of treatment by prednisolone can be confounded with the effects caused by the hospitalization itself. Indeed, most of the sAH patients are malnourished, with their excessive alcohol consumption frequently replacing a standard diet. We cannot exclude that the introduction of the hospital diet combined with alcohol abstinence has effects on the GM. Moreover, most sAH patients included were treated with the combination of prednisolone and N-acetylcysteine: the effects of these two drugs are indiscernible. In addition, an important part of the population was treated with various antibiotics, with well-known effects on the GM, although only a small proportion of GM composition is explained by antibiotics use. Nonetheless, even controlling for antibiotics, we failed to detect a GM signature differing between D0 and D7. Finally, it is possible that a 7-day follow-up is too short a duration for effects on the GM to appear: preclinical studies of the effects of corticosteroids on the GM of rats were conducted over weeks of treatment, rather than days. Moreover, prednisolone is indicated for sAH treatment for a total duration of 28 days. Thus, a longer follow-up would be needed to further study the effects of the therapy on the GM. This may be difficult to achieve since a high proportion of sAH patients are quickly lost to follow-up, with some individuals leaving the hospital only days after presentation.

To conclude, using shotgun metagenomics sequencing, we showed that 7 days of prednisolone therapy have little to no effects on the GM. These results support the relevance of FMT as a treatment to durably reshape GM and ultimately reduce liver inflammation through gut-liver axis modulation. We also found promising results suggesting two BA as potential biomarker to distinguish PR from PNR patients. Further studies with a longer follow-up and including more patients are needed to confirm these findings.

## Highlights

- Prednisolone has little effect on the sAH Gut Microbiota over 7 days
- sAH Gut Microbiota varies by individual rather than by time to treatment
- The ratio of glyco-conjugated to tauro-conjugated Bile Acids significantly changes after 7 days
- Two Bile Acids were significantly enriched in non-responder patients
- Serum Lipopolysaccharide-Binding Protein concentration is correlated to prednisolone response

## Supplementary Material

Supplemental Material

## Data Availability

The sequencing files and the associated metadata are available under the BioProject accession number PRJNA1104421. A temporary link is available for reviewing at the following address https://dataview.ncbi.nlm.nih.gov/object/PRJNA1104421?reviewer=n0cdqve0273ta7798racgkg3ec.
