# Supplementary material for: Serum bile acids profiles are altered without change of the gut microbiota composition following a seven-day prednisolone therapy in severe alcoholic hepatitis
Source: Gut Microbes. 2024 Jul 30;16(1):2382767. doi: 10.1080/19490976.2024.2382767 (PMC11290774; doi:10.1080/19490976.2024.2382767)
Supplement: Supplemental Material [file KGMI_A_2382767_SM7614.zip › Supplementary_Article_DEsparteiro.docx]

**Serum bile acids profiles are altered without change of the gut microbiota composition following a seven-day prednisolone therapy in severe alcoholic hepatitis**

Damien Esparteiro^1^, Grégory Fouquet^1^, Anoïsia Courtois^1^, Guillaume Jedraszak^2^, Léa Marticho^3^, Mathilde Gourdel^4^, Stéphanie Billon-Crossouard^4^, Mickaël Croyal^4^, Mickaël Naassila^1^, Eric Nguyen-Khac^1,3^, Ingrid Marcq^1^

1: GRAP INSERM U1247, Amiens, France

2: Service de Génétique, CHU d’Amiens, France

3: Service d’Hépato-Gastro-Entérologie, CHU d’Amiens, France

4: UMS 016 INSERM, CRNH-Ouest Mass Spectrometry Core Facility, Nantes, France

**Table of contents**

- **Fig. S1. Evaluation of the required sequencing depth.**
- **Table S1. Evolution of blood biochemical markers between D0 and D7 of treatment**
- **Table S2. Species only detected at D0**
- **Table S3. Species only detected at D7**
- **Table S4. Results of the multivariate microbial abundance analysis**.
- **Table S5. Evolution of fecal Short Chain Fatty Acids concentrations (µmol/g) for PR and PNR patients between D0 and D7 of treatment.**
- **Table S6. Evolution of fecal bile acids concentrations (µg/mL) for PR and PNR patients between D0 and D7 of treatment.**
- **Fig. S2. Bacterial relative abundances in stool samples of sAH patients after taxonomic assignment**.

**Fig. S1. Evaluation of the required sequencing depth.**


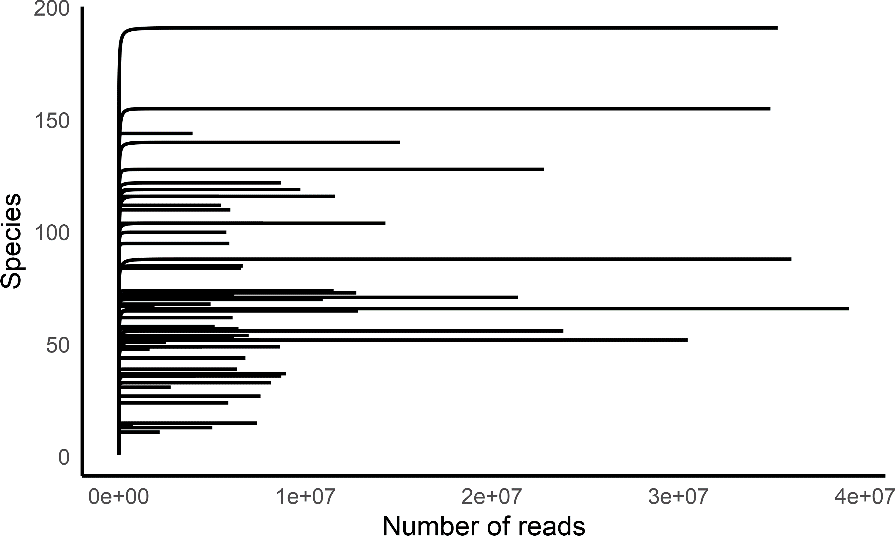


The sequencing depth was sufficiently high to capture most of the species diversity, in spite of an important library size variation.

**Table S1. Evolution of blood biochemical markers between D0 and D7 of treatment.**

|  | **PR (n=16) PNR (n=11)** | **Test** |  |
| --- | --- | --- | --- |
|  | **PR-D0 PR-D7 PNR-D0 PNR-D7** | **Statistic** | **P-value** |
| Albumin (g/L) | 27.3 ± 5.1 29.0 ± 7.8 22.5 ± 5.1 24.5 ± 5.8 | H = 4.896 | 0.180 |
| Alkaline phosphatase (IU/L) | 155.9 ± 32.5 175.1 ± 87.3 163.8 ± 40.9 183.8 ± 82.5 | H = 0.762 | 0.859 |
| Alanine aminotransferase (IU/L) | 43.8 ± 20.9^a^ 57.9 ± 23.3^ab^ 61.0 ± 29.2^ab^ 82.2 ± 40.4^b^ | H = 10.677 | **0.014** |
| Aspartate aminotransferase (IU/L) | 110.2 ± 39.4 110.2 ± 38.8 141.3 ± 105.8 160.5 ± 85.2 | H = 1.912 | 0.591 |
| Creatinin (µmol/L) | 92.9 ± 97.6 70.2 ± 45.9 58.3 ± 12.4 65.9 ± 21.7 | H = 0.317 | 0.957 |
| C-reactive protein (mg/L) | 42.1 ± 29.7 24.3 ± 15.1 28.7 ± 19.0 25.6 ± 16.9 | F = 2.162 | 0.104 |
| γ-glutamyltransferase (IU/L) | 212.6 ± 154.3 219.4 ± 154.6 265.5 ± 210.1 208.7 ± 117.9 | H = 2.228 | 0.973 |
| Prothrombin time (%) | 41.4 ± 9.1 45.7 ± 8.9 36.3 ± 10.7 35.9 ± 12.3 | F = 2.853 | **0.047** |
| Total bilirubin (µmol/L) | 225.7 ± 140.6 168.4 ± 128.9 246.4 ± 133.0 280.5 ± 112.7 | H = 6.814 | 0.078 |
| White cell count (x10^9^/L) | 11.1 ± 11.0 12.7 ± 11.1 8.7 ± 4.7 11.8 ± 5.8 | H = 3.198 | 0.362 |
| Lymphocytes (% of white cell count) | 15.0 ± 6.9 15.6 ± 8.9 12.5 ± 5.9 10.8 ± 5.2 | F = 1.205 | 0.318 |
| Monocytes (% of white cell count) | 9.6 ± 2.3 7.5 ± 2.1 9.4 ± 3.2 8.6 ± 3.0 | F = 2.035 | 0.121 |
| Polymorphonuclear basophils (% of white cell count) | 0.8 ± 0.8 0.4 ± 0.6 0.4 ± 0.7 0.2 ± 0.7 | H = 5.946 | 0.114 |
| Polymorphonuclear eosinophils (% of white cell count) | 2.1 ± 1.4 1.8 ± 1.1 1.5 ± 1.0 1.4 ± 1.3 | F = 0.964 | 0.417 |
| Polymorphonuclear neutrophils (% of white cell count) | 72.0 ± 8.2 74.8 ± 11.4 76.4 ± 8.4 78.4 ± 8.1 | F = 1.090 | 0.362 |

Values are presented as mean ± standard-deviation. p-values < 0.05 are bolded and statistically significant. Depending on the variable distribution, ANOVA F or Kruskal-Wallis H statistics were calculated. Letters figured in exponent denote a significant difference as assessed by post-hoc tests. D0: Day 0 of the treatment; D7: Day 7 of the treatment.

**Table S2. Species only detected at D0**

| Taxa | Relative Abundance (%) | Prevalence (%) | Detection frequency |
| --- | --- | --- | --- |
| s Anaeroglobus_geminatus | 0.048701077 | 18.52 | 5 / 27 |
| s Actinomyces_SGB17154 | 0.080696525 | 11.11 | 3 / 27 |
| s Porphyromonas_pasteri | 0.057712661 | 11.11 | 3 / 27 |
| s Solobacterium_SGB6833 | 0.012609501 | 11.11 | 3 / 27 |

| s Haemophilus_parahaemolyticus | 0.508374878 | 11.11 | 3 / 27 |
| --- | --- | --- | --- |
| s Rothia_dentocariosa | 0.020042842 | 7.407 | 2 / 27 |
| s Propionibacterium_freudenreichii | 0.035082448 | 7.407 | 2 / 27 |
| s Lancefieldella_rimae | 0.069139193 | 7.407 | 2 / 27 |
| s Olsenella_sp_oral_taxon_807 | 0.066155542 | 7.407 | 2 / 27 |
| s Slackia_piriformis | 0.070038516 | 7.407 | 2 / 27 |
| s Bacteroidales_bacterium | 0.108545862 | 7.407 | 2 / 27 |
| s Prevotella_denticola | 0.023038329 | 7.407 | 2 / 27 |
| s Prevotella_veroralis | 0.028971336 | 7.407 | 2 / 27 |
| s Candidatus_Saccharibacteria_unclassified_SGB19782 | 0.011258781 | 7.407 | 2 / 27 |
| s Leuconostoc_gelidum | 0.010180334 | 7.407 | 2 / 27 |
| s GGB9237_SGB14179 | 0.009269884 | 7.407 | 2 / 27 |
| s Clostridium_sp_NSJ_42 | 0.08849797 | 7.407 | 2 / 27 |
| s Clostridium_sp_SN20 | 0.010028162 | 7.407 | 2 / 27 |
| s Mogibacterium_timidum | 0.033243089 | 7.407 | 2 / 27 |
| s Blautia_stercoris | 0.168130855 | 7.407 | 2 / 27 |
| s GGB3463_SGB4621 | 0.006052454 | 7.407 | 2 / 27 |
| s Lachnoanaerobaculum_orale | 0.098128395 | 7.407 | 2 / 27 |
| s Oribacterium_parvum | 0.005893526 | 7.407 | 2 / 27 |
| s Shuttleworthia_satelles | 0.0117451 | 7.407 | 2 / 27 |
| s Stomatobaculum_longum | 0.024336961 | 7.407 | 2 / 27 |
| s Faecalicoccus_pleomorphus | 0.03424544 | 7.407 | 2 / 27 |
| s Holdemania_filiformis | 0.003595383 | 7.407 | 2 / 27 |
| s Colibacter_massiliensis | 0.076726348 | 7.407 | 2 / 27 |
| s Neisseria_subflava | 0.152459412 | 7.407 | 2 / 27 |
| s Actinomyces_dentalis | 0.027193503 | 3.704 | 1 / 27 |
| s Actinomyces_johnsonii | 0.005229105 | 3.704 | 1 / 27 |
| s Actinomyces_naeslundii | 0.182560949 | 3.704 | 1 / 27 |
| s Actinomyces_timonensis | 0.01318138 | 3.704 | 1 / 27 |
| s Bifidobacterium_pullorum | 0.003152395 | 3.704 | 1 / 27 |
| s Lawsonella_SGB3665 | 0.315562586 | 3.704 | 1 / 27 |
| s Atopobium_minutum | 0.04668955 | 3.704 | 1 / 27 |
| s Olsenella_provencensis | 0.010507371 | 3.704 | 1 / 27 |
| s Olsenella_uli | 0.06899471 | 3.704 | 1 / 27 |
| s Collinsella_intestinalis | 0.007320077 | 3.704 | 1 / 27 |
| s Collinsella_massiliensis | 0.00429403 | 3.704 | 1 / 27 |
| s GGB45432_SGB63101 | 0.006631608 | 3.704 | 1 / 27 |
| s GGB9361_SGB14336 | 0.016112673 | 3.704 | 1 / 27 |
| s GGB1355_SGB1819 | 0.036318778 | 3.704 | 1 / 27 |
| s Phocaeicola_barnesiae | 1.624654108 | 3.704 | 1 / 27 |
| s Porphyromonas_SGB1980 | 0.022595218 | 3.704 | 1 / 27 |
| s Porphyromonas_asaccharolytica | 0.022237097 | 3.704 | 1 / 27 |
| s Porphyromonas_endodontalis | 0.061755649 | 3.704 | 1 / 27 |
| s Porphyromonas_gingivalis | 0.139650519 | 3.704 | 1 / 27 |
| s Porphyromonas_sp_oral_taxon_278 | 0.064214225 | 3.704 | 1 / 27 |

| s Alloprevotella_tannerae | 0.021998683 | 3.704 | 1 / 27 |
| --- | --- | --- | --- |
| s Prevotella_aurantiaca | 0.067118256 | 3.704 | 1 / 27 |
| s Prevotella_corporis | 0.088825283 | 3.704 | 1 / 27 |
| s Prevotella_koreensis | 0.006159289 | 3.704 | 1 / 27 |
| s Prevotella_multiformis | 0.009261921 | 3.704 | 1 / 27 |
| s Prevotella_nanceiensis | 0.052743728 | 3.704 | 1 / 27 |
| s Prevotella_nigrescens | 0.074725278 | 3.704 | 1 / 27 |
| s Prevotella_oris | 0.014477135 | 3.704 | 1 / 27 |
| s Prevotella_pallens | 0.007132309 | 3.704 | 1 / 27 |
| s Prevotella_salivae | 0.008997294 | 3.704 | 1 / 27 |
| s Alistipes_provencensis | 0.06780382 | 3.704 | 1 / 27 |
| s Parabacteroides_gordonii | 0.48246748 | 3.704 | 1 / 27 |
| s Tannerella_forsythia | 0.006579045 | 3.704 | 1 / 27 |
| s GGB1109_SGB1423 | 11.8806696 | 3.704 | 1 / 27 |
| s GGB1203_SGB1568 | 0.011616127 | 3.704 | 1 / 27 |
| s Capnocytophaga_gingivalis | 0.028928954 | 3.704 | 1 / 27 |
| s Capnocytophaga_leadbetteri | 0.010459654 | 3.704 | 1 / 27 |
| s Candidatus_Gastranaerophilales_bacterium | 1.240056092 | 3.704 | 1 / 27 |
| s Candidatus_Nanosynsacchari_sp_TM7_ANC_38_39_G1_1 | 0.026881568 | 3.704 | 1 / 27 |
| s Gemella_morbillorum | 0.005645015 | 3.704 | 1 / 27 |
| s Staphylococcus_epidermidis | 6.3015722 | 3.704 | 1 / 27 |
| s Staphylococcus_haemolyticus | 8.874844253 | 3.704 | 1 / 27 |
| s Bacilli_unclassified_SGB6493 | 0.072311834 | 3.704 | 1 / 27 |
| s Abiotrophia_defectiva | 0.004980839 | 3.704 | 1 / 27 |
| s Granulicatella_adiacens | 0.005122718 | 3.704 | 1 / 27 |
| s Granulicatella_elegans | 0.001717631 | 3.704 | 1 / 27 |
| s Enterococcus_sp_63_4 | 0.008699207 | 3.704 | 1 / 27 |
| s Lactobacillus_acidophilus | 0.007037161 | 3.704 | 1 / 27 |
| s Lentilactobacillus_parabuchneri | 0.105890797 | 3.704 | 1 / 27 |
| s Pediococcus_acidilactici | 0.011778805 | 3.704 | 1 / 27 |
| s Pediococcus_pentosaceus | 0.114563683 | 3.704 | 1 / 27 |
| s Streptococcus_rubneri | 0.053017854 | 3.704 | 1 / 27 |
| s Streptococcus_sobrinus | 0.004290349 | 3.704 | 1 / 27 |
| s GGB2945_SGB3918 | 0.008550147 | 3.704 | 1 / 27 |
| s GGB35456_SGB47582 | 0.030298211 | 3.704 | 1 / 27 |
| s GGB9581_SGB79823 | 0.046134645 | 3.704 | 1 / 27 |
| s GGB9522_SGB14921 | 0.022437487 | 3.704 | 1 / 27 |
| s Clostridium_SGB6179 | 0.15753072 | 3.704 | 1 / 27 |
| s Clostridium_neonatale | 0.034454173 | 3.704 | 1 / 27 |
| s GGB9574_SGB14987 | 0.00155176 | 3.704 | 1 / 27 |
| s GGB9580_SGB14998 | 0.01040057 | 3.704 | 1 / 27 |
| s Clostridiales_bacterium_Marseille_P5551 | 0.007253838 | 3.704 | 1 / 27 |
| s Lawsonibacter_sp_NSJ_51 | 0.011448238 | 3.704 | 1 / 27 |
| s Bariatricus_massiliensis | 0.056623203 | 3.704 | 1 / 27 |
| s Coprococcus_sp_AF21_14LB | 0.003390576 | 3.704 | 1 / 27 |

| s GGB3394_SGB4498 | 0.01037399 | 3.704 | 1 / 27 |
| --- | --- | --- | --- |
| s GGB3424_SGB4551 | 0.02038268 | 3.704 | 1 / 27 |
| s GGB3887_SGB5270 | 0.003377136 | 3.704 | 1 / 27 |
| s Lachnoanaerobaculum_sp_ICM7 | 0.011282035 | 3.704 | 1 / 27 |
| s Lachnospiraceae_unclassified_SGB66069 | 0.025300577 | 3.704 | 1 / 27 |
| s Lacrimispora_SGB36971 | 9.477967032 | 3.704 | 1 / 27 |
| s Lacrimispora_saccharolytica | 0.130411092 | 3.704 | 1 / 27 |
| s Oribacterium_SGB5283 | 0.005593902 | 3.704 | 1 / 27 |
| s Oribacterium_sinus | 0.015565268 | 3.704 | 1 / 27 |
| s GGB9618_SGB15065 | 0.007855218 | 3.704 | 1 / 27 |
| s GGB9739_SGB15313 | 0.232192851 | 3.704 | 1 / 27 |
| s Acidaminococcus_massiliensis | 0.008048177 | 3.704 | 1 / 27 |
| s Selenomonas_SGB5880 | 0.009022497 | 3.704 | 1 / 27 |
| s Selenomonas_sputigena | 0.012680645 | 3.704 | 1 / 27 |
| s Megasphaera_sp_AM44_1BH | 0.107716468 | 3.704 | 1 / 27 |
| s Megasphaera_sp_MJR8396C | 12.73783904 | 3.704 | 1 / 27 |
| s Negativicoccus_succinicivorans | 0.008154196 | 3.704 | 1 / 27 |
| s Veillonella_sp_3627 | 0.021587493 | 3.704 | 1 / 27 |
| s Parvimonas_SGB6649 | 0.006313485 | 3.704 | 1 / 27 |
| s Parvimonas_micra | 0.004514527 | 3.704 | 1 / 27 |
| s Parvimonas_sp_KA00067 | 0.025005922 | 3.704 | 1 / 27 |
| s Peptoniphilus_lacrimalis | 0.005389935 | 3.704 | 1 / 27 |
| s Peptoniphilus_sp_Marseille_P3761 | 0.009344729 | 3.704 | 1 / 27 |
| s Fusobacterium_necrophorum | 0.034788305 | 3.704 | 1 / 27 |
| s Leptotrichia_sp_oral_taxon_215 | 0.032072846 | 3.704 | 1 / 27 |
| s Leptotrichia_wadei | 0.015354626 | 3.704 | 1 / 27 |
| s Sneathia_amnii | 0.003393383 | 3.704 | 1 / 27 |
| s Neisseria_cinerea | 0.005482538 | 3.704 | 1 / 27 |
| s Neisseria_elongata | 0.013389385 | 3.704 | 1 / 27 |
| s Neisseria_sicca | 0.409005891 | 3.704 | 1 / 27 |
| s Mailhella_massiliensis | 0.075153296 | 3.704 | 1 / 27 |
| s Campylobacter_SGB19314 | 0.022358475 | 3.704 | 1 / 27 |
| s Succinatimonas_hippei | 0.174383285 | 3.704 | 1 / 27 |
| s Kluyvera_ascorbata | 0.027121544 | 3.704 | 1 / 27 |
| s Morganella_morganii | 0.009032196 | 3.704 | 1 / 27 |
| s Proteus_mirabilis | 0.084405384 | 3.704 | 1 / 27 |
| s Haemophilus_haemolyticus | 0.020694541 | 3.704 | 1 / 27 |
| s Haemophilus_pittmaniae | 0.075191862 | 3.704 | 1 / 27 |
| s Pseudomonas_aeruginosa | 0.390473101 | 3.704 | 1 / 27 |
| s Stenotrophomonas_maltophilia | 0.759310548 | 3.704 | 1 / 27 |
| s Xanthomonas_campestris | 0.181169863 | 3.704 | 1 / 27 |

**Table S3. Species only detected at D7**

| Taxa | Relative Abundance (%) | Prevalence (%) | Detection frequency |
| --- | --- | --- | --- |
| s Olsenella_sp_GAM18 | 0.031723504 | 7.407 | 2 / 27 |
| s Butyricimonas_faecalis | 0.644883698 | 7.407 | 2 / 27 |
| s Enterococcus_gallinarum | 0.026663546 | 7.407 | 2 / 27 |
| s GGB32463_SGB47515 | 0.03505252 | 7.407 | 2 / 27 |
| s GGB4964_SGB6927 | 0.031117055 | 7.407 | 2 / 27 |
| s Clostridium_butyricum | 1.24767491 | 7.407 | 2 / 27 |
| s Blautia_hydrogenotrophica | 0.115175181 | 7.407 | 2 / 27 |
| s Lachnoclostridium_edouardi | 0.021285959 | 7.407 | 2 / 27 |
| s Lachnospiraceae_bacterium_NSJ_29 | 0.115292405 | 7.407 | 2 / 27 |
| s Mediterraneibacter_butyricigenes | 0.01444814 | 7.407 | 2 / 27 |
| s GGB9636_SGB15107 | 0.031822186 | 7.407 | 2 / 27 |
| s Ruminococcaceae_unclassified_SGB15265 | 0.653512507 | 7.407 | 2 / 27 |
| s Faecalitalea_cylindroides | 0.001932831 | 7.407 | 2 / 27 |
| s Megasphaera_hexanoica | 0.0080299 | 7.407 | 2 / 27 |
| s Citrobacter_freundii | 2.859835662 | 7.407 | 2 / 27 |
| s Enterobacter_hormaechei | 0.671056073 | 7.407 | 2 / 27 |
| s Methanosphaera_stadtmanae | 0.013379385 | 3.704 | 1 / 27 |
| s Actinomyces_urogenitalis | 0.191973374 | 3.704 | 1 / 27 |
| s Parolsenella_catena | 0.057087686 | 3.704 | 1 / 27 |
| s Enorma_SGB14753 | 0.00942796 | 3.704 | 1 / 27 |
| s Coriobacteriia_bacterium | 0.00920266 | 3.704 | 1 / 27 |
| s Eggerthellaceae_unclassified_SGB14322 | 0.003125637 | 3.704 | 1 / 27 |
| s Paraeggerthella_hongkongensis | 0.561914594 | 3.704 | 1 / 27 |
| s Raoultibacter_massiliensis | 0.004226914 | 3.704 | 1 / 27 |
| s Bacteroides_sp_Marseille_P3684 | 0.019000878 | 3.704 | 1 / 27 |
| s Sanguibacteroides_justesenii | 0.126826862 | 3.704 | 1 / 27 |
| s GGB1215_SGB1581 | 0.026761228 | 3.704 | 1 / 27 |
| s Prevotella_bivia | 0.028036926 | 3.704 | 1 / 27 |
| s Prevotella_copri_clade_B | 0.163758817 | 3.704 | 1 / 27 |
| s Prevotella_hominis | 0.697605231 | 3.704 | 1 / 27 |
| s Prevotella_pectinovora | 0.063240123 | 3.704 | 1 / 27 |
| s Prevotella_timonensis | 0.066438633 | 3.704 | 1 / 27 |
| s Alistipes_SGB2307 | 0.057574703 | 3.704 | 1 / 27 |
| s GGB1689_SGB2321 | 0.029479707 | 3.704 | 1 / 27 |
| s Rikenellaceae_unclassified_SGB2184 | 0.040588755 | 3.704 | 1 / 27 |
| s Parabacteroides_faecis | 0.001945458 | 3.704 | 1 / 27 |
| s Lentilactobacillus_buchneri | 0.115961681 | 3.704 | 1 / 27 |
| s Lactobacillus_coleohominis | 0.01476833 | 3.704 | 1 / 27 |
| s Streptococcus_massiliensis | 0.009371042 | 3.704 | 1 / 27 |
| s Streptococcus_sanguinis | 0.02174883 | 3.704 | 1 / 27 |
| s GGB8965_SGB13825 | 0.00153116 | 3.704 | 1 / 27 |
| s GGB9186_SGB14125 | 0.010623786 | 3.704 | 1 / 27 |
| s GGB58158_SGB79798 | 0.004062311 | 3.704 | 1 / 27 |

| s GGB45491_SGB63163 | 0.011247695 | 3.704 | 1 / 27 |
| --- | --- | --- | --- |
| s Candidatus_Borkfalkia_ceftriaxoniphila | 0.002445759 | 3.704 | 1 / 27 |
| s Candidatus_Parachristensenella_avicola | 0.00403229 | 3.704 | 1 / 27 |
| s Candidatus_Pararuminococcus_gallinarum | 0.003970012 | 3.704 | 1 / 27 |
| s Clostridia_unclassified_SGB14844 | 0.090085547 | 3.704 | 1 / 27 |
| s Clostridia_unclassified_SGB6385 | 1.114942953 | 3.704 | 1 / 27 |
| s Clostridia_unclassified_SGB71281 | 0.033571972 | 3.704 | 1 / 27 |
| s GGB9176_SGB14114 | 0.045268074 | 3.704 | 1 / 27 |
| s GGB9342_SGB14306 | 0.017677425 | 3.704 | 1 / 27 |
| s GGB9537_SGB14940 | 0.003125766 | 3.704 | 1 / 27 |
| s GGB9787_SGB15410 | 0.015147127 | 3.704 | 1 / 27 |
| s Catabacter_hongkongensis | 0.003588493 | 3.704 | 1 / 27 |
| s Christensenella_minuta | 0.005717629 | 3.704 | 1 / 27 |
| s Clostridium_baratii | 0.224877478 | 3.704 | 1 / 27 |
| s Clostridium_tepidum | 0.281066678 | 3.704 | 1 / 27 |
| s Clostridium_transplantifaecale | 0.093948818 | 3.704 | 1 / 27 |
| s Clostridiales_bacterium_Choco116 | 0.017264332 | 3.704 | 1 / 27 |
| s Clostridiales_bacterium_NSJ_40 | 0.016550923 | 3.704 | 1 / 27 |
| s Massilistercora_timonensis | 0.013812655 | 3.704 | 1 / 27 |
| s Anaerosporobacter_mobilis | 0.097341512 | 3.704 | 1 / 27 |
| s Blautia_schinkii | 0.007275003 | 3.704 | 1 / 27 |
| s Frisingicoccus_caecimuris | 0.135456541 | 3.704 | 1 / 27 |
| s GGB3614_SGB4886 | 0.093742347 | 3.704 | 1 / 27 |
| s GGB3678_SGB4991 | 0.002956831 | 3.704 | 1 / 27 |
| s Tyzzerella_sp_An114 | 0.027964399 | 3.704 | 1 / 27 |
| s Oscillibacter_SGB15077 | 0.050068708 | 3.704 | 1 / 27 |
| s Anaerotruncus_massiliensis | 0.072980038 | 3.704 | 1 / 27 |
| s Angelakisella_massiliensis | 0.008609116 | 3.704 | 1 / 27 |
| s Bittarella_massiliensis | 0.004800736 | 3.704 | 1 / 27 |
| s GGB9501_SGB14898 | 0.020363701 | 3.704 | 1 / 27 |
| s GGB9621_SGB15073 | 0.003290032 | 3.704 | 1 / 27 |
| s GGB9622_SGB15074 | 0.002717597 | 3.704 | 1 / 27 |
| s GGB9640_SGB15115 | 0.018489724 | 3.704 | 1 / 27 |
| s GGB9691_SGB15198 | 0.152671187 | 3.704 | 1 / 27 |
| s GGB9694_SGB15201 | 0.019456276 | 3.704 | 1 / 27 |
| s GGB9694_SGB15203 | 0.034280341 | 3.704 | 1 / 27 |
| s GGB9709_SGB15238 | 0.032962097 | 3.704 | 1 / 27 |
| s Pseudoflavonifractor_SGB15156 | 0.249477 | 3.704 | 1 / 27 |
| s Pseudoflavonifractor_capillosus | 0.017003489 | 3.704 | 1 / 27 |
| s Ruminococcus_SGB4421 | 1.79285202 | 3.704 | 1 / 27 |
| s Clostridium_saccharogumia | 0.019380593 | 3.704 | 1 / 27 |
| s Erysipelotrichaceae_bacterium | 0.056550077 | 3.704 | 1 / 27 |
| s Holdemanella_biformis | 0.094745597 | 3.704 | 1 / 27 |
| s Massilimicrobiota_timonensis | 0.003272662 | 3.704 | 1 / 27 |
| s Firmicutes_bacterium | 0.034418987 | 3.704 | 1 / 27 |

| s GGB4262_SGB5800 | 0.422119147 | 3.704 | 1 / 27 |
| --- | --- | --- | --- |
| s Megasphaera_massiliensis | 0.012040817 | 3.704 | 1 / 27 |
| s Leptotrichia_hongkongensis | 0.019656584 | 3.704 | 1 / 27 |
| s Alphaproteobacteria_bacterium | 0.021796754 | 3.704 | 1 / 27 |
| s GGB6649_SGB9391 | 0.01970513 | 3.704 | 1 / 27 |
| s Parasutterella_SGB9260 | 0.039296249 | 3.704 | 1 / 27 |
| s GGB6544_SGB9243 | 0.008734728 | 3.704 | 1 / 27 |
| s GGB6546_SGB9245 | 0.01646405 | 3.704 | 1 / 27 |
| s GGB6547_SGB9247 | 0.021923474 | 3.704 | 1 / 27 |
| s Desulfovibrio_fairfieldensis | 0.056366772 | 3.704 | 1 / 27 |
| s Campylobacter_curvus | 1.076670018 | 3.704 | 1 / 27 |
| s Klebsiella_grimontii | 0.135007563 | 3.704 | 1 / 27 |
| s Klebsiella_michiganensis | 0.408144055 | 3.704 | 1 / 27 |
| s Hafnia_paralvei | 0.101255673 | 3.704 | 1 / 27 |
| s Haemophilus_sputorum | 0.068959981 | 3.704 | 1 / 27 |
| s Pseudomonas_fragi | 0.396943585 | 3.704 | 1 / 27 |
| s Pseudomonas_helleri | 0.104999684 | 3.704 | 1 / 27 |
| s Pseudomonas_weihenstephanensis | 0.023893313 | 3.704 | 1 / 27 |
| s Cloacibacillus_evryensis | 0.087191301 | 3.704 | 1 / 27 |
| s Pyramidobacter_piscolens | 0.118460273 | 3.704 | 1 / 27 |

**Table S4. Results of the multivariate microbial abundance analysis**.

Associations between microbial abundances and individual features were tested using multivariate linear mixed model in MaAsLin 2 with “Time” (D0 vs D7), Sex, Age, Response and Antibiotics variables as fixed effect, and Individual as random effect. Only the features with a prevalence of 10 % and above and with an absolute read count equal to or higher than 500 were tested. P-values were corrected using the Benjamini-Hochberg procedure.

**Table S5. Evolution of fecal Short Chain Fatty Acids concentrations (µmol/g) for PR and PNR patients between D0 and D7 of treatment.**

**PR (n=8) PNR (n=9) Test**

|  | **D0** | **D7** | **D0** | **D7** | **Statistic** | **P-value** |
| --- | --- | --- | --- | --- | --- | --- |
| **Total SCFA (µmol/g)** | 94.6 ± 72.2 | 112.4 ± 34.1 | 115.8 ± 63.4 | 145.4 ± 54.6 | F = 1.134 | 0.351 |
| Acetate (% of total) | 79.3 ± 12.3 | 81.6 ± 12.7 | 77.0 ± 13.8 | 82.5 ± 10.4 | F = 0.356 | 0.785 |
| Propionate (% of total) | 11.1 ± 8.7 | 8.8 ± 8.1 | 12.7 ± 9.1 | 10.3 ± 5.9 | F = 0.339 | 0.797 |
| Butyrate (% of total) | 8.1 ± 5.8 | 7.0 ± 4.3 | 7.9 ± 5.8 | 5.9 ± 4.5 | H = 2.075 | 0.557 |
| Isobutyrate (% of total) | 0.4 ± 0.6 | 0.9 ± 1.3 | 0.9 ± 1.3 | 0.4 ± 0.4 | H = 1.913 | 0.591 |
| Valerate (% of total) | 0.7 ± 0.9 | 0.8 ± 1.3 | 0.8 ± 1.0 | 0.4 ± 0.7 | F = 1.648 | 0.649 |
| Isovalerate (% of total) | 0.5 ± 0.6 | 0.8 ± 1.0 | 0.7 ± 0.6 | 0.5 ± 0.4 | H = 1.655 | 0.647 |

Values are presented as mean ± standard-deviation. Depending on the variable distribution, ANOVA F or Kruskal-Wallis H statistics were calculated. D0:

Day 0 of the treatment; D7: Day 7 of the treatment; PNR: Prednisolone Non- Responders; PR: Prednisolone Responders

**Table S6. Evolution of stool samples bile acids concentrations for PR and PNR patients between D0 and D7 of treatment.**

|  | | **PR (n=8)** | |  | **PNR (n=6)** | | **Test** | |
| --- | --- | --- | --- | --- | --- | --- | --- | --- |
| **D0** | |  | | **D7** | **D0 D7** | | **Statistic P-value** | |
| **Total bile acids (µg/g)** 174.7 ± 185.3 116.5 ± 145.2 32.5 ± 26.5 65.2 ± 107.5 H = 4.516 0.211 | | | | | | | | |
| **Primary bile acids (% of total)** | 62.9 ± 38.7 | | 55.7 ± 45.6 | | 55.8 ± 37.9 | 62.0 ± 31.5 | H = 0.228 | 0.973 |
| **Free bile acids (% of total)** | 93.1 ± 6.3 | | 96.0 ± 7.1 | | 86.2 ± 17.8 | 93.9 ± 3.0 | H = 4.465 | 0.215 |
| CA (% of total) | 35.7 ± 29.7 | | 38.3 ± 38.8 | | 24.6 ± 25.5 | 32.6 ± 23.8 | H = 1.912 | 0.912 |
| CDCA (% of total) | 20.8 ± 12.8 | | 13.8 ± 14.1 | | 18.3 ± 10.2 | 23.3 ± 11.6 | H = 2.669 | 0.446 |
| DCA (% of total) | 17.2 ± 18.3 | | 18.7 ± 21.7 | | 10.3 ± 9.6 | 11.0 ± 12.2 | H = 0.567 | 0.904 |
| HDCA (% of total) | 1.0 ± 2.0 | | 0.6 ± 1.4 | | 0.5 ± 1.1 | 0.4 ± 0.9 | H = 0.652 | 0.884 |
| LCA (% of total) | 14.0 ± 17.0 | | 23.3 ± 27.5 | | 26.7 ± 31.7 | 12.5 ± 18.1 | H = 1.511 | 0.680 |
| UDCA (% of total) | 4.5 ± 4.8 | | 1.3 ± 1.9 | | 5.8 ± 5.0 | 14.0 ± 13.7 | H = 5.267 | 0.153 |
| **Glycoconjugated bile acids (% of total)** | 6.9 ± 6.3 | | 4.0 ± 7.1 | | 13.8 ± 17.8 | 6.1 ± 3.0 | H = 4.465 | 0.215 |
| CA (% of total) | 1.9 ± 3.1 | | 1.2 ± 3.0 | | 2.9 ± 5.9 | 0.1 ± 0.3 | H = 2.444 | 0.485 |
| CDCA (% of total) | 4.6 ± 4.2 | | 2.3 ± 3.7 | | 10.1 ± 12.3 | 5.9 ± 3.1 | H = 5.853 | 0.119 |
| DCA (% of total) | 0.3 ± 0.5 | | 0.3 ± 0.6 | | 0.2 ± 0.5 | 0.1 ± 0.2 | H = 0.567 | 0.904 |
| HDCA (% of total) | 0.0 ± 0.0 | | 0.0 ± 0.0 | | 0.0 ± 0.0 | 0.0 ± 0.0 | - | - |
| LCA (% of total) | 0.1 ± 0.3 | | 0.1 ± 0.2 | | 0.5 ± 1.1 | 0.0 ± 0.0 | H = 1.673 | 0.643 |
| UDCA (% of total) | 0.0 ± 0.0 | | 0.0 ± 0.0 | | 0.3 ± 0.6 | 0.0 ± 0.0 | H = 3.667 | 0.300 |

Values are presented as mean ± standard-deviation. p-values < 0.05 are bolded and statistically significant. Kruskal-Wallis H statistic was calculated.

Abbreviations: CA: Cholic Acid; CDCA: Chenodeoxycholic Acid; DCA: Deoxycholic Acid; D0: Day 0 of the treatment; D7: Day 7 of the treatment; HDCA: Hyodeoxycholic Acid; LCA: Lithocholic Acid; PNR, prednisolone non-responders; PR, prednisolone responders; UDCA: Ursodeoxycholic Acid.


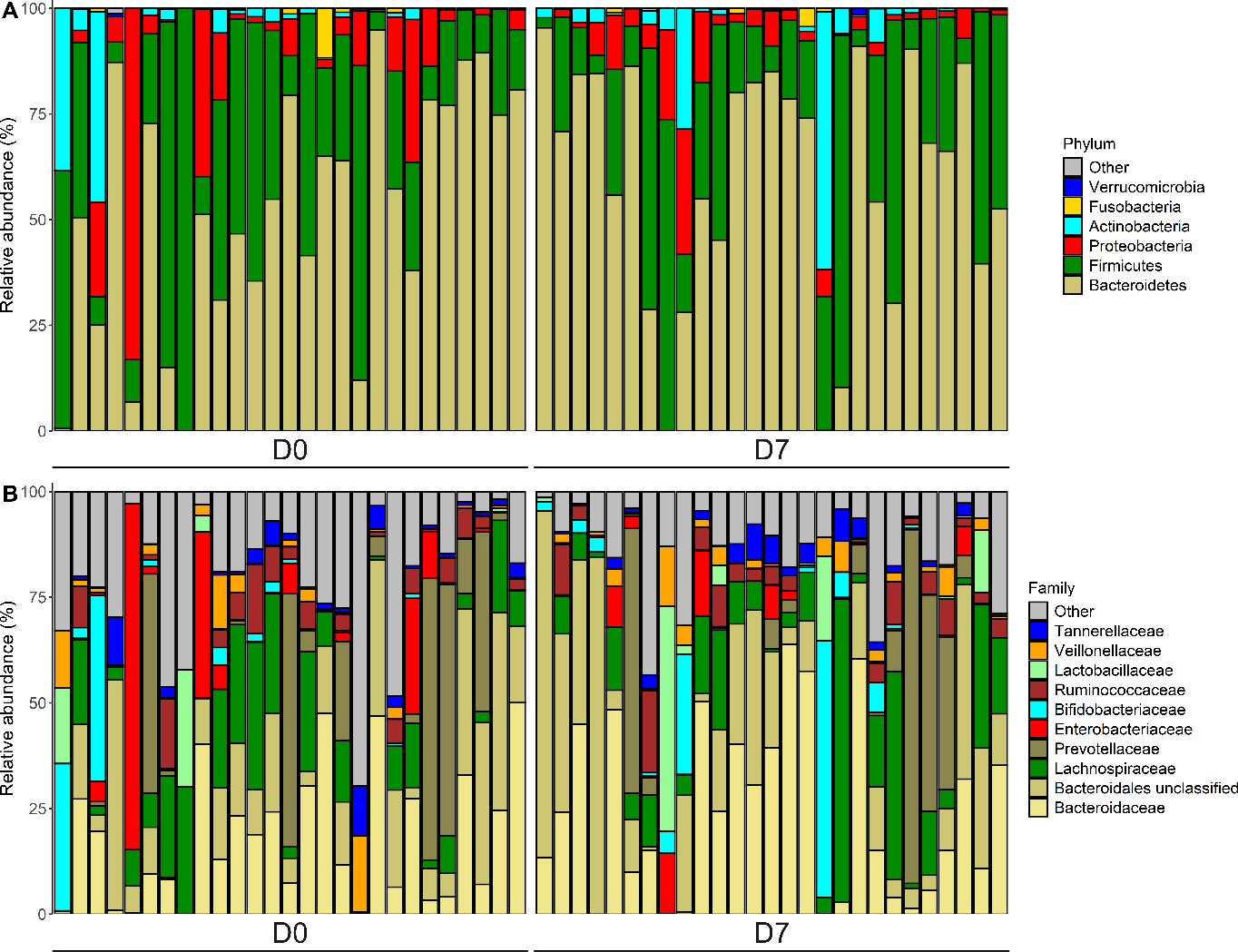
**Fig.S2. Bacterial relative abundances in stool samples of sAH patients after taxonomic assignment**.

No significant change was observed between D0 and D7 either at the phylum

(A) or family (B) levels.
